# Regulation of Inflammatory Pathways in Cancer and Infectious Disease of the Cervix

**DOI:** 10.6064/2012/548150

**Published:** 2012-05-30

**Authors:** Anthonio Adefuye, Kurt Sales

**Affiliations:** MRC/UCT Research Group for Receptor Biology, Institute of Infectious Disease and Molecular Medicine and Division of Medical Biochemistry, Faculty of Health Sciences, University of Cape Town, Observatory, Cape Town, 7925, South Africa

## Abstract

Cervical cancer is one of the leading gynaecological malignancies worldwide. It is an infectious disease of the cervix, associated with human papillomavirus infection (HPV), infection with bacterial agents such as *Chlamydia trachomatis* and *Neisseria gonorrhoea* as well as human immunodeficiency virus (HIV). Furthermore, it is an AIDS-defining disease with an accelerated mortality in HIV-infected women with cervical cancer. With the introduction of robust vaccination strategies against HPV in the developed world, it is anticipated that the incidence of cervical cancer will decrease in the coming years. However, vaccination has limited benefit for women already infected with high-risk HPV, and alternative therapeutic intervention strategies are needed for these women. Many pathological disorders, including cervical cancer, are characterised by the exacerbated activation and maintenance of inflammatory pathways which are considered to be regulated by infectious agents. In cervical cancer, hyperactivation of these inflammatory pathways and regulation of immune infiltrate into tissues can potentially play a role not only in tumorigenesis but also in HIV infection. In this paper we will discuss the contribution of inflammatory pathways to cervical cancer progression and HIV infection and the role of HIV in cervical cancer progression.

## 1. Introduction

Cervical cancer is the most common gynaecological cancer among women in developing countries [1, 2]. Virtually all cases of cervical cancer follow after infection of the cervical epithelium with oncogenic human papilloma virus (HPV) types [3]. Currently, there are over 150 genotypes of HPV [4]. These are species-specific and tissue-tropic and only infect cutaneous or internal squamous mucosal surfaces in humans [4, 5]. Around 40 types are known to infect the anogenital tract, giving rise to genital warts, condylomata or cancers, and their precursor lesions [4]. The majority of anogenital cancers in humans are associated with the high-risk HPV16 and 18 and there is correlation between percentage of HPV 16 and 18 integration and severity of the cervical lesions [6]. Although it is necessary to have infection of the cervix with oncogenic HPV to develop cancer, HPV itself may not be sufficient. Other associated cofactors including compromised immune system or infections with herpes virus II [7], *Chlamydia trachomatis* [8], *Neisseria gonorrhoeae* [9], or bacterial vaginosis [10] have been associated with cervical inflammation and increased risk of cervical cancer. In 1993, cervical cancer was classified as an AIDS-defining disease, together with Kaposi Sarcoma and Non-Hodgkin Lymphoma, in women infected with human immunodeficiency virus (HIV) [11]. This highlighted HIV as a potent cofactor for developing invasive carcinoma of the cervix and highlighted cervical cancer as an infectious disease. Although HPV infection is very common among young sexually active women, only a small percentage of women below the age of 25 years actually develop cervical cancer. In fact, the median age recently reported for women presenting with invasive cervical cancer is around 50 years of age [12, 13]. These observations highlight the long latency of the virus and the need for persistent infection of the cervix to promote disease.

**Figure 1:**
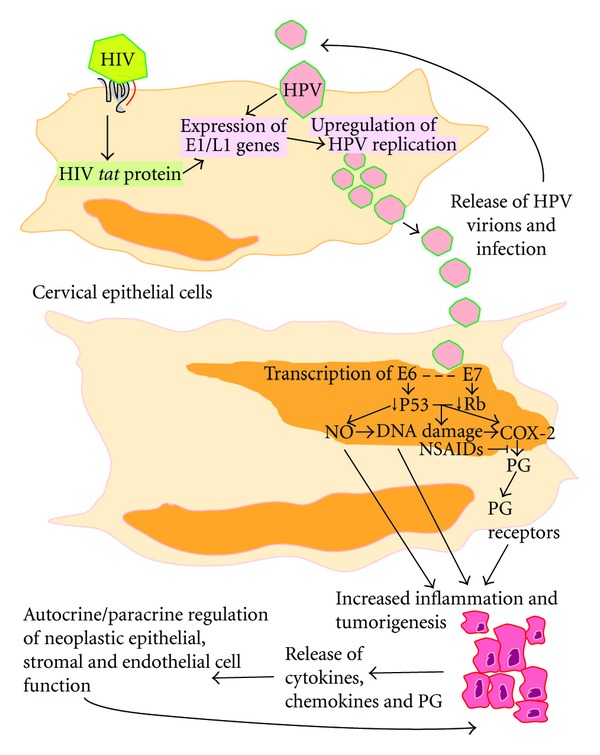
HIV infects cells and the tat protein causes the amplification of the HPV E1/L1 genes leading to increased HPV replication and release of HPV virions. These then infect the same or adjacent cervical epithelial cell. Within the epithelial cells, the HPV E6 oncoprotein binds to P53 protein targeting it for ubiquitin-dependent degradation while the E7 binds to the Rb protein thus disrupting the Rb and E2F complex and can increase nitric oxide (NO) production, DNA damage and the activation of the COX-2/PG/PG receptor inflammatory pathways leading to increased inflammation and tumorigenesis. Inflammatory and tumor cells can then release cytokines, chemokines and PG which act in autocrine/paracrine to regulate the endothelial, stromal, neoplastic epithelial and infiltrating immune cells function to cause an increased tumour angiogenesis, increased tumour growth, decreased apoptosis, and decreased local immune-surveillance. These conditions favour tumorigenesis and viral survival in the tumor microenvironment. Inhibition of the COX-PG cascade with nonsteroidal anti-inflammatory drugs (NSAIDs) like aspirin could reduce inflammation and tumour progression by inhibiting downstream pathways activated by PG and promote proresolution by elevating expression of resolution lipid mediators such as Resolvins.

 HPV enters the body and infects basal keratinocytes, exposed through mild abrasion or microtrauma to the cervico-vaginal epithelium (Figure 1). The main route of HPV transmission is via exposure of the cervix to virus present in saliva or seminal fluid or in the effected partner's skin during coitus. After transmission, the virus remains in the epithelial mucosa, where it is hidden from contact with the bloodstream and the innate immune system. HPV thus manages to evade immune detection and immune-evasion is considered to be a key aspect of HPV persistence [5]. Even though there is no viraemia or cytolysis associated with initial viral infection of the cervix, and no activation of the innate immune system and inflammation, the virus actively induces mechanisms to evade immune detection and ensure its success by deregulating the interferon pathway and via the down-regulation of pattern recognition receptors such as Toll-like receptor 9, thereby allowing infection to proceed undetected for a considerable time [5]. Within the basal cells, the HPV viral oncogenes are incorporated into the host's DNA, where they induce viral DNA synthesis, using the host's DNA replication machinery. Viral replication and protein synthesis, which in large amounts would elicit an immune response, are low in the basal layers. However, as the basal cells mature, differentiate, and migrate towards the surface epidermal layer, rapid DNA synthesis commences. When the cells reach the superficial layers of the epidermis, they undergo natural apoptosis and release the viral particles—a process which takes about 3 weeks. Once detected, the innate and adaptive immune systems initiate an inflammatory response against the virus, effectively eliminating it in the majority of infected individuals [10, 14]. However, approximately 15% of women fail to clear the virus [15, 16] leading to persistent infection, which together with inadequate resolution or exacerbation of activation of inflammatory pathways can promote malignant progression [5, 17]. In this review, we will discuss some of the molecular pathways activated in the cervix by infection with HPV and HIV and the relevance of these to inflammation and cancer.

## 2. Inflammatory Pathways

Inflammation involves a coordinate effort by the body to maintain homeostasis in the face of insult, by bacterial or viral pathogen, or by injury. The process of inflammation is highly complex involving a host of resident and recruited cell types, which work together to promote the removal of the insult or injury and initiate the repair of the tissue (reviewed in [18]). When successful, this results in the restoration of tissue homeostasis, a process termed resolution (reviewed in [19–21]).

### 2.1. Inflammatory Cell Component of Tumours

The inflammatory infiltrate of tumours can comprise a vast population of different immune cells at any one time—each capable of producing cytokines or other factors which can alter the fate of immune populations within the tumour. The inflammatory environment in tumours is characterised by the presence of leukocytes. Leukocytes are recruited into the tissue in response to cytokines and chemokines released by tumour cells and are resident both in the supporting stroma as well as the tumour itself [22]. Polymorphonuclear leukocytes (PMN's or neutrophils) are the first immune cell types to be recruited to sites of inflammation, followed by monocytes which are derived from haemopoietic progenitor cells [23]. When in the tissue, monocytes then differentiate into either macrophages or dendritic cells (called Langerhans cells in the epidermis). Langerhans cells generally constitute the first defence against pathogens. Recently, tumour associated neutrophils have been described that are capable of polarising the phenotype of other immune cells and altering the cellular composition of the tumour microenvironment [24]. Here, PMNs are thought to exist in either an N1 state, capable of killing tumour cells by producing and releasing cytotoxic compounds, or an N2 state capable of promoting tumour growth by modulating the cytokine/chemokine environment in the tumour [24]. A significant proportion of the immune infiltrate in tumours is comprised of tumour-associated macrophages (TAMs). These are derived from circulating monocyte precursors via the release of monocyte chemotactic protein (MCP) chemokines [17]. Although TAMs are a heterogeneous cell population, early stage tumours are thought have type 1 macrophages (M1). These produce proinflammatory cytokines and chemokines, such as CXCL10 and CXCL19 to recruit Th1, Th17, and natural killer (NK) cells [25]. In more advanced tumours, TAMs polarise towards a type 2 (M2) state to encourage Th2 differentiation and the production of potent angiogenic factors such as VEGF to facilitate tissue remodelling and tumorigenesis [26].

### 2.2. Cyclooxygenase-Prostaglandin Pathway

The inflammatory cyclooxygenase-(COX-) prostaglandin (PG) axis is a central pathway regulating inflammation and cancer (reviewed in detail in [27, 28]). There are two COX enzyme isoforms in humans, namely, COX-1 and COX-2 [29]. The role of COX enzymes in inflammation has been underscored by studies involving the use of nonsteroidal anti-inflammatory drugs (NSAIDs), which function by blocking COX-derived lipid mediators, namely, prostaglandins, prostacyclins and thromboxanes [30]. *In vitro* and *in vivo* studies have highlighted a role of COX-derived lipid mediators in driving tissue remodelling events associated with inflammation. More recently, NSAIDs have been shown to induce active proresolution pathways to facilitate healing, by acetylating COX-2 enzyme and altering its enzymatic capacity to produce classes of proresolution lipid mediators such as Resolvins (reviewed in [18–20]).

### 2.3. Cytokines

The cytokine and chemoattractive cytokine (chemokine) networks vary in many tumour tissues, compared with normal tissue [31], and have been recently reviewed [32]. One of the main differences in tissue remodelling between tumour and normal tissue is the elevated level of hypoxia often present in tumours, which induces the release of cytokines and chemokines [33]. Tumour necrosis factor (TNF) is a major cytokine involved in inflammation and tissue remodelling by stimulating fibroblast growth and angiogenesis as well as the production of MCP, which regulates the infiltration of macrophages and lymphocytes [17]. Inflammatory cytokines such as interleukin (IL) 1 and IL-6 stimulate cell growth and tumour development and play a role in metastases. IL-1 also plays a role in leukocyte adherence to the vascular endothelium, to promote extravasation of the vasculature and tumour infiltration [17]. IL-6 promotes the activation of the coagulation cascade, differentiation of B cells, and T cell activation [34]. Inflammatory cytokines are also major inducers of chemokines in many resident and recruited cell types. Chemokines are grouped on the basis of the arrangement of the two N-terminal cysteine residues and are designated as CC, if the cysteine residues are adjacent to one another or CXC if first two cysteine residues have an amino acid between them. CXC chemokines are generally active on neutrophils and lymphocytes, whereas CC chemokines act on monocytes, dendritic cells, lymphocytes and natural killer (NK) cells, but not neutrophils [14].

## 3. HPV Oncogenes: Structure and Role in Inflammation and Tumorigenesis

The HPV genome consists of 3 domains, a non-coding upstream regulatory region, an early region containing open reading frames E6, E7, E1, E2, E4, and E5 and a late region encoding the major (L1) and minor (L2) capsid proteins [5]. Once infected, the E6 and E7 early genes are actively transcribed and the E6 and E7 oncoproteins, both of which are involved in neoplastic transformation, are actively expressed [35]. Although E6 and E7 oncogenes appear to be the main HPV genes involved in transformation, recent studies have highlighted a role for E5 oncogene in immune cell modulation and tumorigenesis [36] and regulation of late viral functions together with E4 oncogene. E1 and E2 oncogenes encode replication factors and are thought to play a role in HPV persistence by allowing episomal copies of the virus to be maintained in the nucleus and partitioned into daughter cells during mitosis [37]. Many of the observations on the roles of HPV oncogenes have been derived using *in vitro* model systems of HPV-containing cell lines where specific oncogenes have been ablated by RNA interference, or HPV-negative cells that have been transfected with cDNA constructs containing specific HPV oncogenes. These studies have shown that both oncoproteins target different molecular pathways in the cell. E6 oncoprotein binds to P53 protein and targets it for ubiquitin-dependent degradation, whereas E7 binds to family members of the retinoblastoma protein (Rb) and disrupts the complex between Rb and the E2F transcription factor family (Figure 1) [38]. This facilitates immortalisation of cells and triggers the early steps in malignant conversion by interfering with cell cycle control [5, 38]. The result is uncontrolled replication and a buildup of damaged DNA—ideal conditions for cancer development.

Animal studies using transgenic mouse models of cervical cancer have highlighted more specifically the contribution of individual oncogenes to tumorigenesis and have shown that the E7 protein induces high-grade cervical dysplasia and invasive tumours, whereas the E6 protein only induces low-grade cervical dysplasia. Furthermore, they have highlighted that both oncogenes work in synergy to promote cervical cancer progression since coexpression of E6 and E7 proteins produce larger and more extensive tumours compared with E7 alone [39].

### 3.1. Regulation of Cervical Inflammation by HPV Oncogenes

The direct association between HPV infection and inflammation is still a topic of debate, however, studies by Subbaramaiah and Dannenberg and Oh and colleagues have now emerged to show that in neoplastic cervical epithelial cells, HPV 16 E5, E6, and E7 oncogenes can induce the inflammatory COX-PG axis, by elevating expression of the immediate early oncogene COX-2 and expression of the E-series prostaglandin receptors (PTGER) such as PTGER2 and PTGER4 (Figure 1) [36, 40, 41]. Many chronic inflammatory diseases, allergy, asthma, atherosclerosis, autoimmunity, transplant rejection, metabolic, and degenerative diseases and cancer [42] are all associated with upregulation in COX enzyme expression. In the past decade, several studies have emerged from *in vitro* and *in vivo* model systems employing cell lines and rodents to demonstrate that prostaglandins, produced as a consequence of elevated COX enzyme expression, can promote extensive tissue remodelling within tumours by evoking all the classical hallmarks of cancer, namely, cellular proliferation, angiogenesis, inhibition of apoptosis, and alteration in vascular permeability to allow immune cell extravasation from the vasculature [18, 43]. The studies of Subbaramaiah and Dannenberg and Oh and colleagues, described above, for the first time demonstrate that these hallmarks of cancer can be driven by HPV oncogenes [36, 40, 41] and provide a direct link between HPV oncogenes and activation of potent inflammatory cascades, with known roles in promoting chronic inflammation and cancer.

### 3.2. Inflammatory Pathways Regulated by HPV Oncogenes and COX Enzymes

Inflammation involves extensive tissue remodelling events which are orchestrated by complex networks of cytokines, chemokines, and bioactive lipids. These work across multiple cellular compartments to elicit their function. The classic hallmarks of inflammation are the recruitment of immune cells into the tissue and alteration of vascular function to allow for immune cell extravasation. This alteration in permeability causes oedema and the redness generally associated with tissue inflammation. Several reports have correlated inflammatory cell infiltrate with HPV-induced high-grade lesions. Infiltrating lymphocytes are thought to contribute to tumour growth and spread as well as immunosuppression, generally associated with malignant diseases [14]. Although the precise mechanism whereby HPV oncogenes regulate tissue remodelling events is unclear, HPV infections have been shown to promote the release of inflammatory mediators and cytokines from keratinocytes to alter the immune response and promote the infiltration of macrophages, lymphocytes and NK cells (reviewed in [44]). Changes in the vasculature to facilitate immune extravasation and angiogenesis require tissue remodelling of the extracellular matrix, a process facilitated by matrix metalloproteinase (MMP) [13]. Several studies have correlated HPV E6 and E7 transcription with MMP transcription [45, 46] and genes in cervical epithelial cells involved in tissue differentiation and remodelling [47]. In addition, transfection studies have shown that E7 oncoprotein forms a complex with and downregulates leukocyte elastase inhibitor [48]. This would facilitate the activation of neutrophils and promote neutrophil influx into the tissue. Furthermore, transgenic mouse models where the early region genes from HPV16 are expressed under the control of the human keratin 14 promoter have shown that macrophage recruitment to HPV-associated lesions occurs via the release of the chemokine CCL2 and interaction with its receptor CCR2 present on macrophages [49].

Once resident in the tissue, inflammatory cells are known to produce vast amounts of reactive oxygen species and nitric oxide which have been shown to induce DNA damage (Figure 1) [50] and to contribute towards the progression of the disease in high-grade cervical lesions [50]. Furthermore, nitric oxide has been shown to induce transcription of E6 and E7 oncogenes in cervical epithelial cells [51], which can further enhance inflammation via an autoamplifying positive feedback loop via activation of COX-2 and other parallel inflammatory pathways. Taken together, these studies provide compelling evidence for a role for HPV oncogenes in regulation of inflammation in cervical cancers. It is tempting to speculate that targeted inhibition of E6 and E7 actions in cervical epithelial cells could be a potential therapeutic alternative to vaccination for women infected with HPV.

## 4. HIV, Cervical Cancer, and Inflammation

The human immunodeficiency virus (HIV); the only etiological factor attributed to the acquired immunodeficiency syndrome (AIDS) belongs to the genus Lentivirus within the family *Retroviridae* [52]. About 33 million people harbour this virus worldwide [53] with high epidemic rates in sub-Saharan Africa. Mature HIV virion is spherical (approximate diameter of 100–120 nm), with a genome of two copies of identical (9.2kb) single-stranded RNA [54]. HIV is divided into two main subtypes HIV-1 and HIV-2. HIV-1 is further subtyped into phylogenetically related clades; types A-K, with subtype C being the most common type in Africa [55].

Transmission is via unprotected sexual intercourse, intravenous drug use, blood transfusion, infection with blood-derived products, or mother-to-foetal transmission. To initiate infection, virus attaches to cellular surfaces via an interaction between the gp120 viral envelope protein and a receptor complex present on the host cell consisting of the CD4 receptor and G protein-coupled receptor (GPCR) coreceptor, usually CCR5 or CXCR4 [54]. Most primary HIV-1 variants are restricted to the use of CCR5 and CXCR4 [56, 57], however, they have been shown to use alternative receptors *in vitro*. HIV-2 variants are capable of infecting a wider range of cells expressing different coreceptors such as GPR15 and CXCR6 in addition to CCR5 and CXCR4 [58]. This leads to fusion between the viral and cellular membranes and ultimate release of the viral core into the cell cytoplasm [54]. Once inside the host cell the virus is reversed transcribed to full-length double-stranded DNA by the reverse transcriptase enzyme and is integrated into the host genome [59]. The hallmark of infection is characterised by progressive depletion of CD4^+^ T-cells leading to an immunodeficiency state, paving the way for opportunistic infection and ultimately mortality [60].

### 4.1. The Interplay between HIV and HPV and Their Role in Cervical Cancer

The interplay between HPV and HIV is complex; however, their synergistic role in exacerbating pathology of the cervix has been well documented. For example, epidemiological studies have shown that women that are coinfected by HPV and HIV have an estimated 41 fold increase in the risk of developing neoplastic cervical lesions [61] and HIV infected immune-compromised women have been shown to have a higher prevalence of HPV-induced lesions [62]. Furthermore, studies in Sub-Saharan women, where 67% of the population are living with HIV/AIDS, have shown that women with HIV develop cervical cancer at an earlier age than women who are HIV negative [63–65].

Although the precise mechanisms predisposing women infected with HPV to HIV infection are unclear, there is evidence that clearance of HPV infection from the female genital tract elicits a cell-mediated immune response characterised by gross infiltration of lymphocytes and macrophages into the epithelium [66], which can enhance the risk of HIV infecting immune cells in the cervix in these women after unprotected sexual contact.

Central to the role of HIV in cervical cancer is its ability to ablate the systemic immune response to infection, including HPV infection. This can facilitate inadequate clearance of HPV in infected individuals, enhancing HPV persistence or reinfection, and it increases the likelihood that precancerous lesions will develop into cancer. To this end, HIV may modify HPV-related carcinogenesis by altering the expression of inflammatory components (cytokines) in the cervix and diminution of local cervical cellular immunity, thus altering HPV regulation [53]. For example, HIV *tat *gene has been shown to increase the expression of HPV E1 and L1 genes, hence causing upregulation of the HPV replication (Figure 1) [67, 68]. Furthermore, HIV-1 tat protein is capable of transactivating HPV16 transcription [69].

### 4.2. The Role of HIV in Regulating Inflammation and Its Potential Contribution to Cervical Cancer

Systemic expression of several proinflammatory cytokines has been reported to be a major feature in HIV infection. The virus causes immune dysregulation leading to increase in the production of proinflammtory cytokines such as TNF*α*, IL-1, and IL-6 which are detected in the plasma and lymph node of infected patient [70]. This link between HIV and production of pro-inflammatory cytokine was suggested by the observation that the virus and/or its surface glycoprotein gp120 can induce *in vitro* secretion of TNF, IL-1, and IL-6 by monocytes isolated from uninfected individuals [71–73]. Supplementary studies also detected high levels of IL-1*α*, IL-1*β*, IL-6, and TNF in the serum and cerebrospinal fluid of seropositive individuals [74, 75]. Often associated with the production of these cytokines is the elevated secretion of CC-chemokines such as macrophage inflammatory protein (MIP)-1*α*, MIP-1*β*, and RANTES [76, 77]. These proinflammatory cytokines expressed as soluble factors or membrane binding molecules and are directly or indirectly involved in HIV entry and T cell apoptosis [60]. These cytokines have been found to be abundant in microenvironment of several tumours including cervical cancer where they are secreted by the tumour cells, endothelial cells, and/or infiltrating activated immune cells where they act as endogenous tumour promoter by stimulating the production of transcription factors (e.g., NF-*κ*B, AP-1), proliferative and angiogenic proteins (e.g., VEGF, MMP), and adhesion molecules (e.g., E-selectin, VCAM), thus enhancing tumour growth and mediating tumour metastasis [78]. TNF, a major mediator of inflammation, can be detected in various human neoplasias where it is implicated in the induction of MCP-1 which can modulate the infiltration of immune cells in to the tumour microenvironment. It is, therefore, feasible that the elevated level of these proinflammatory mediators in HIV infected individuals can drive tumour progression in HIV-positive women with cervical dysplasia.

In addition to regulation of immune cell infiltrate, alteration in the expression profile of HIV receptors on cells within the cervico-vaginal region could impact on HIV acquisition and cervical cancer progression. The epithelial surface of the female reproductive tract expresses all the receptors necessary for HIV infection including CD4, CCR5, and CXCR4 [79]. Maher and colleagues have recently shown that HIV virions can bind the external surface of cervical epithelium and penetrate beneath the epithelial surface [80]. Thus, it is plausible that epithelial cells lining the cervico-vaginal interface could be the first cells to come into contact with HIV and might play a role in the replication of the virus and transmission to leukocytes present in the submucosa.

Expression of some of the HIV receptors on uterine epithelial cells display a temporal variation in expression during the menstrual cycle, indicating that they are hormonally regulated [79]. This could alter susceptibility to infection depending on the phase of the menstrual cycle. Chemokine receptors such as CXCR4 are elevated in cervical cancer and play a role in lymph node metastasis during advanced-stage disease [81]. These receptors can also be hijacked by HIV for entry in such women. CXCR4 expression can be regulated by HPV oncogenes [82] and prostaglandins [83] in the female genital tract. These observations suggest that HPV infection and inflammation can drive expression of HIV coreceptors on epithelial cells. It is unknown whether the inflammatory milieu of cervical cancer can also alter expression of HIV receptors on immune cells in the tumour periphery. It is plausible that in women with HPV infection or localised cervico-vaginal inflammation, alterations in HIV receptor expression could allow more virus to bind the epithelium and elevate the amount of virus present locally in the genital tract following intercourse. In women with cervical cancers or inflammatory cell infiltrate into the cervix, this could enhance susceptibility to infection.

## 5. Concluding Remarks

It is clear that inflammation plays a critically important role in regulating pathology of cervix, susceptibility to infection by virus, like HPV and HIV and patient outcome. The grand challenge that lies ahead for the developing world, where a significant proportion of women live with either HPV or HIV infection or both, is how to manage inflammation in these women to prevent disease progression, especially progression of cervical cancer. Clinical trials have shown that long-term treatment with low-dose nonsteroidal anti-inflammatory drugs (NSAIDS) like aspirin can be beneficial for reducing the burden of colorectal cancer [84]. In resource poor countries, it is tempting to speculate that low-dose aspirin, which is affordable and widely available, could be of benefit to women with HPV infections and neoplastic lesions, by suppressing the inflammatory COX-PG axis, promoting resolution of inflammation, and preventing progression of cervical cancer.

## References

[1] Arbyn M, Castellsague X, de Sanjose S (2011). Worldwide burden of cervical cancer in 2008. *Annals of Oncology*.

[2] Anorlu RI (2008). Cervical cancer: the sub-Saharan African perspective. *Reproductive Health Matters*.

[3] zur Hausen H (2009). Papillomaviruses in the causation of human cancers—a brief historical account. *Virology*.

[4] Carter JR, Ding Z, Rose BR (2011). HPV infection and cervical disease: a review. *Australian and New Zealand Journal of Obstetrics and Gynaecology*.

[5] Stanley MA, Pett MR, Coleman N (2007). HPV: from infection to cancer. *Biochemical Society Transactions*.

[6] Cañadas MP, Videla S, Darwich L (2010). Human papillomavirus HPV-16, 18, 52 and 58 integration in cervical cells of HIV-1-infected women. *Journal of Clinical Virology*.

[7] Smith JS, Herrero R, Bosetti C (2002). Herpes simplex virus-2 as a human papillomavirus cofactor in the etiology of invasive cervical cancer. *Journal of the National Cancer Institute*.

[8] Anttila T, Saikku P, Koskela P (2001). Serotypes of Chlamydia trachomatis and risk for development of cervical squamous cell carcinoma. *Journal of the American Medical Association*.

[9] Castle PE, Giuliano AR (2003). Genital tract infections, cervical inflammation, and antioxidant nutrients—assessing their roles as human papillomavirus cofactors. *Journal of the National Cancer Institute*.

[10] Castle PE, Hillier SL, Rabe LK (2001). An association of cervical inflammation with high-grade cervical neoplasia in women infected with oncogenic human papillomavirus (HPV). *Cancer Epidemiology Biomarkers and Prevention*.

[11] Levine AM (1993). AIDS-related malignancies: the emerging epidemic. *Journal of the National Cancer Institute*.

[12] de Sanjose S, Quint WGV, Alemany L (2010). Human papillomavirus genotype attribution in invasive cervical cancer: a retrospective cross-sectional worldwide study. *The Lancet Oncology*.

[13] Libra M, Scalisi A, Vella N (2009). Uterine cervical carcinoma: role of matrix metalloproteinases. *International Journal of Oncology*.

[14] Balkwill F, Mantovani A (2001). Inflammation and cancer: back to Virchow?. *The Lancet*.

[15] Frazer IH (2009). Interaction of human papillomaviruses with the host immune system: a well evolved relationship. *Virology*.

[16] Scott M, Nakagawa M, Moscicki AB (2001). Cell-mediated immune response to human papillomavirus infection. *Clinical and Diagnostic Laboratory Immunology*.

[17] Coussens LM, Werb Z (2002). Inflammation and cancer. *Nature*.

[18] Jabbour HN, Sales KJ, Catalano RD, Norman JE (2009). Inflammatory pathways in female reproductive health and disease. *Reproduction*.

[19] Hutchinson JL, Rajagopal SP, Sales KJ, Jabbour HN (2011). Molecular regulators of resolution of inflammation: potential therapeutic targets in the reproductive system. *Reproduction*.

[20] Serhan CN, Brain SD, Buckley CD (2007). Resolution of inflammation: state of the art, definitions and terms. *The FASEB Journal*.

[21] Serhan CN, Chiang N, Van Dyke TE (2008). Resolving inflammation: dual anti-inflammatory and pro-resolution lipid mediators. *Nature Reviews Immunology*.

[22] Negus RPM, Stamp GWH, Hadley J, Balkwill FR (1997). Quantitative assessment of the leukocyte infiltrate in ovarian cancer and its relationship to the expression of C-C chemokines. *American Journal of Pathology*.

[23] Ziegler-Heitbrock L (2007). The CD14+ CD16+ blood monocytes: their role in infection and inflammation. *Journal of Leukocyte Biology*.

[24] Houghton AM (2010). The paradox of tumor-associated neutrophils: fueling tumor growth with cytotoxic substances. *Cell Cycle*.

[25] Krausgruber T, Blazek K, Smallie T (2011). IRF5 promotes inflammatory macrophage polarization and T H1-TH17 responses. *Nature Immunology*.

[26] Biswas SK, Mantovani A (2010). Macrophage plasticity and interaction with lymphocyte subsets: cancer as a paradigm. *Nature Immunology*.

[27] Rizzo MT (2011). Cyclooxygenase-2 in oncogenesis. *Clinica Chimica Acta*.

[28] Smith WL, DeWitt DL, Garavito RM (2000). Cyclooxygenases: structural, cellular, and molecular biology. *Annual Review of Biochemistry*.

[29] Vane JR, Bakhle YS, Botting RM (1998). Cyclooxygenases 1 and 2. *Annual Review of Pharmacology and Toxicology*.

[30] Vane JR, Botting RM (1998). Anti-inflammatory drugs and their mechanism of action. *Inflammation Research*.

[31] Burke F, Relf M, Negus R, Balkwill F (1996). A cytokine profile of normal and malignant ovary. *Cytokine*.

[32] Lazennec G, Richmond A (2010). Chemokines and chemokine receptors: new insights into cancer-related inflammation. *Trends in Molecular Medicine*.

[33] Koong AC, Denko NC, Hudson KM (2000). Candidate genes for the hypoxic tumor phenotype. *Cancer Research*.

[34] Guazzone VA, Jacobo P, Theas MS, Lustig L (2009). Cytokines and chemokines in testicular inflammation: a brief review. *Microscopy Research and Technique*.

[35] Middleton K, Peh W, Southern S (2003). Organization of human papillomavirus productive cycle during neoplastic progression provides a basis for selection of diagnostic markers. *Journal of Virology*.

[36] Oh JM, Kim SH, Lee YI (2009). Human papillomavirus E5 protein induces expression of the EP4 subtype of prostaglandin E2 receptor in cyclic AMP response element-dependent pathways in cervical cancer cells. *Carcinogenesis*.

[37] Bodily J, Laimins LA (2011). Persistence of human papillomavirus infection: keys to malignant progression. *Trends in Microbiology*.

[38] Dyson N, Guida P, Munger K, Harlow E (1992). Homologous sequences in adenovirus E1A and human papillomavirus E7 proteins mediate interaction with the same set of cellular proteins. *Journal of Virology*.

[39] Riley RR, Duensing S, Brake T, Münger K, Lambert PF, Arbeit JM (2003). Dissection of human papillomavirus E6 and E7 function in transgenic mouse models of cervical carcinogenesis. *Cancer Research*.

[40] Subbaramaiah K, Dannenberg AJ (2007). Cyclooxygenase-2 transcription is regulated by human papillomavirus 16 E6 and E7 oncoproteins: evidence of a corepressor/coactivator exchange. *Cancer Research*.

[41] Oh JM, Kim SH, Cho EA, Song YS, Kim WH, Juhnn YS (2010). Human papillomavirus type 16 E5 protein inhibits hydrogen peroxide-induced apoptosis by stimulating ubiquitin-proteasome-mediated degradation of Bax in human cervical cancer cells. *Carcinogenesis*.

[42] Wymann MP, Schneiter R (2008). Lipid signalling in disease. *Nature Reviews Molecular Cell Biology*.

[43] Sales KJ, Jabbour HN (2003). Cyclooxygenase enzymes and prostaglandins in pathology of the endometrium. *Reproduction*.

[44] Boccardo E, Lepique AP, Villa LL (2010). The role of inflammation in HPV carcinogenesis. *Carcinogenesis*.

[45] Nuovo GJ, MacConnell PB, Simsir A, Valea F, French DL (1995). Correlation of the in situ detection of polymerase chain reaction- amplified metalloproteinase complementary DNAs and their inhibitors with prognosis in cervical carcinoma. *Cancer Research*.

[46] da Silva Cardeal LB, Brohem CA, Corrêa TCS (2006). Higher expression and activity of metalloproteinases in human cervical carcinoma cell lines is associated with HPV presence. *Biochemistry and Cell Biology*.

[47] Duffy CL, Phillips SL, Klingelhutz AJ (2003). Microarray analysis identifies differentiation-associated genes regulated by human papillomavirus type 16 E6. *Virology*.

[48] Lee KA, Kang JW, Shim JH (2005). Protein profiling and identification of modulators regulated by human papillomavirus 16 E7 oncogene in HaCaT keratinocytes by proteomics. *Gynecologic Oncology*.

[49] Pahler JC, Tazzyman S, Erez N (2008). Plasticity in tumor-promoting inflammation: impairment of macrophage recruitment evokes a compensatory neutrophil response. *Neoplasia*.

[50] Hiraku Y, Tabata T, Ma N, Murata M, Ding X, Kawanishi S (2007). Nitrative and oxidative DNA damage in cervical intraepithelial neoplasia associated with human papilloma virus infection. *Cancer Science*.

[51] Wei L, Gravitt PE, Song H, Maldonado AM, Ozbun MA (2009). Nitric oxide induces early viral transcription coincident with increased DNA damage and mutation rates in human papillomavirus-infected cells. *Cancer Research*.

[52] Girard MP, Osmanov S, Assossou OM, Kieny MP (2011). Human immunodeficiency virus (HIV) immunopathogenesis and vaccine development: a review. *Vaccine*.

[53] Pantanowitz L, Michelow P (2011). Review of human immunodeficiency virus (HIV) and squamous lesions of the uterine cervix. *Diagnostic Cytopathology*.

[54] Sierra S, Kupfer B, Kaiser R (2005). Basics of the virology of HIV-1 and its replication. *Journal of Clinical Virology*.

[55] Taylor BS, Sobieszczyk ME, McCutchan FE, Hammer SM (2008). The challenge of HIV-1 subtype diversity. *The New England Journal of Medicine*.

[56] Doranz BJ, Lu ZH, Rucker J (1997). Two distinct ccr5 domains can mediate coreceptor usage by human immunodeficiency virus type 1. *Journal of Virology*.

[57] Shimizu N, Tanaka A, Oue A (2009). Broad usage spectrum of G protein-coupled receptors as coreceptors by primary isolates of HIV. *AIDS*.

[58] Blaak H, Boers PHM, Gruters RA, Schuitemaker H, Van Der Ende ME, Osterhaus ADME (2005). CCR5, GPR15, and CXCR6 are major coreceptors of human immunodeficiency virus type 2 variants isolated from individuals with and without plasma viremia. *Journal of Virology*.

[59] Harrich D, Hooker B (2002). Mechanistic aspects of HIV-1 reverse transcription initiation. *Reviews in Medical Virology*.

[60] Decrion AZ, Dichamp I, Varin A, Herbein G (2005). HIV and inflammation. *Current HIV Research*.

[61] Moodley JR, Hoffman M, Carrara H (2006). HIV and pre-neoplastic and neoplastic lesions of the cervix in South Africa: a case-control study. *BMC Cancer*.

[62] Palefsky JM, Holly EA (2003). Immunosuppression and co-infection with HIV. *Journal of the National Cancer Institute*.

[63] Gichangi PB, Bwayo J, Estambale B (2003). Impact of HIV infection on invasive cervical cancer in Kenyan women. *AIDS*.

[64] Moodley M, Moodley J, Kleinschmidt I (2001). Invasive cervical cancer and human immunodeficiency virus (HIV) infection: a South African perspective. *International Journal of Gynecological Cancer*.

[65] Chirenje ZM (2005). HIV and cancer of the cervix. *Best Practice & Research Clinical Obstetrics & Gynaecology*.

[66] Coleman N, Birley HDL, Renton AM (1994). Immunological events in regressing genital warts. *American Journal of Clinical Pathology*.

[67] Spinillo A, Tenti P, Zappatore R, De Seta F, Silini E, Guaschino S (1993). Langerhans’ cell counts and cervical intraepithelial neoplasia in women with human immunodeficiency virus infection. *Gynecologic Oncology*.

[68] Palefsky J (2006). Biology of HPV in HIV infection. *Advances in Dental Research*.

[69] Vernon SD, Hart CE, Reeves WC, Icenogle JP (1993). The HIV-1 tat protein enhances E2-dependent human papillomavirus 16 transcription. *Virus Research*.

[70] Emilie D, Peuchmaur M, Maillot MC (1990). Production of interleukins in human immunodeficiency virus-1-replicating lymph nodes. *Journal of Clinical Investigation*.

[71] Choe W, Volsky DJ, Potash MJ (2001). Induction of rapid and extensive *β*-chemokine synthesis in macrophages by human immunodeficiency virus type 1 and gp120, independently of their coreceptor phenotype. *Journal of Virology*.

[72] Clouse KA, Cosentino LM, Weih KA (1991). The HIV-1 gp120 envelope protein has the intrinsic capacity to stimulate monokine secretion. *Journal of Immunology*.

[73] Herbein G, Keshav S, Collin M, Montaner LJ, Gordon S (1994). HIV-1 induces tumour necrosis factor and IL-1 gene expression in primary human macrophages independent of productive infection. *Clinical and Experimental Immunology*.

[74] Weiss L, Haeffner-Cavaillon N, Laude M, Gilquin J, Kazatchkine MD (1989). HIV infection is associated with the spontaneous production of interleukin-1 (IL-1) in vivo and with an abnormal release of IL-1*α* in vitro. *AIDS*.

[75] Fauci AS (1996). Host factors in the pathogenesis of HIV disease. *Antibiotics and Chemotherapy*.

[76] Canque B, Rosenzwajg M, Gey A, Tartour E, Fridman WH, Gluckman JC (1996). Macrophage inflammatory protein-1*α* is induced by human immunodeficiency virus infection of monocyte-derived macrophages. *Blood*.

[77] Cotter RL, Zheng J, Che M (2001). Regulation of human immunodeficiency virus type 1 infection, *β*-chemokine production, and CCR5 expression in CD40L-stimulated macrophages: immune control of viral entry. *Journal of Virology*.

[78] Lewis AM, Varghese S, Xu H, Alexander HR (2006). Interleukin-1 and cancer progression: the emerging role of interleukin-1 receptor antagonist as a novel therapeutic agent in cancer treatment. *Journal of Translational Medicine*.

[79] Yeaman GR, Howell AL, Weldon S (2003). Human immunodeficiency virus receptor and coreceptor expression on human uterine epithelial cells: regulation of expression during the menstrual cycle and implications for human immunodeficiency virus infection. *Immunology*.

[80] Maher D, Wu X, Schacker T, Horbul J, Southern P (2005). HIV binding, penetration, and primary infection in human cervicovaginal tissue. *Proceedings of the National Academy of Sciences of the United States of America*.

[81] Kodama J, Hasengaowa, Kusumoto T (2007). Association of CXCR4 and CCR7 chemokine receptor expression and lymph node metastasis in human cervical cancer. *Annals of Oncology*.

[82] Amine A, Rivera S, Opolon P (2009). Novel anti-metastatic action of cidofovir mediated by inhibition of E6/E7, CXCR4 and Rho/ROCK signaling in HPV+ tumor cells. *PLoS ONE*.

[83] Sales KJ, Grant V, Catalano RD, Jabbour HN (2011). Chorionic gonadotrophin regulates CXCR4 expression in human endometrium via E-series prostanoid receptor 2 signalling to PI3K-ERK1/2: implications for fetal-maternal crosstalk for embryo implantation. *Molecular Human Reproduction*.

[84] Burn J, Gerdes AM, Macrae F (2011). Long-term effect of aspirin on cancer risk in carriers of hereditary colorectal cancer: an analysis from the CAPP2 randomised controlled trial. *The Lancet*.

